# The accuracy and timeliness of a Point Of Care lactate measurement in patients with Sepsis

**DOI:** 10.1186/s13049-015-0151-x

**Published:** 2015-09-17

**Authors:** Fatene Ismail, William G. Mackay, Andrew Kerry, Harry Staines, Kevin D. Rooney

**Affiliations:** Institute of Healthcare Policy and Practice, School of Health, Nursing and Midwifery, University of the West of Scotland, Paisley, UK; Institute of Healthcare Associated Infection, University of the West of Scotland and University Hospital Crosshouse, Kilmarnock, UK; Biochemistry Laboratory, Royal Alexandra Hospital, Paisley, UK; Sigma Statistical Services, Balmullo, UK; Intensive Care Unit, Royal Alexandra Hospital and Institute of Healthcare Policy and Practice, University of the West of Scotland, Paisley, UK

## Abstract

**Background:**

The aims of this study were to a) compare the lactate measurement of a Point of Care (POC) handheld device to near patient blood gas analysers, and b) determine the differential reporting times between the analysers.

**Methods:**

A two-staged study; method comparison and prospective observational stages, was conducted. For the first stage, blood samples were analysed on the i-STAT handheld device and the near patient blood gas analysers (GEM 4000 and OMNI S). Results were compared using Pearson correlation coefficient and Bland-Altman tests. For the second stage, we examined the differential reporting times of the POC device compared to the near patient blood gas analysers in two Scottish hospitals. Differential reporting times were assessed using Mann–Whitney test and descriptive statistics were reported with quartiles.

**Results:**

Highly significant Pearson correlation coefficients (0.999 and 0.993 respectively) were found between i-STAT and GEM 4000 and OMNI S. The Bland-Altman agreement method showed bias values of −0.03 and −0.24, between i-STAT and GEM 4000 and OMNI S respectively. Median time from blood draw to i-STAT lactate results was 5 min (Q1–Q3 5–7). Median time from blood draw to GEM 4000 lactate results was 10 min (Q1–Q3 7.75–13). Median time from blood draw to OMNIS lactate results was 11 min (Q1–Q3 8–22). The i-STAT was significantly quicker than both the GEM 4000 and the OMNIS (each p-value < 0.001). In addition, 18 of our study samples were sent to the central laboratory for analysis due to a defect in the lactate module of OMNI S. The median time for these samples from blood draw to availability of the central laboratory results at the clinical area was 133 min.

**Conclusions:**

The POC handheld device produced accurate, efficient and timely lactate measurements with the potential to influence clinical decision making sooner.

## Introduction

Blood lactate is a useful biomarker to identify patients at increased risk of mortality from sepsis. It is also an independent predictor of mortality and critical care admission [1, 2]. Elevated blood lactate is associated with mortality among critically ill patients [2, 3]. Early lactate clearance is associated with a decreased mortality rate in patients with severe sepsis [4]. Persistent elevation of blood lactate of greater than 48 h in haemodynamically stable postoperative patients has been shown to be associated with an increased mortality rate [5]. Repeated and timely monitoring is therefore important in the management of patients with sepsis and in those with an elevated lactate.

Lactate is measured using various analytical approaches including central laboratory methods, near patient blood gas analysers and portable Point Of Care (POC) handheld devices. The central laboratory approach involves transportation of blood samples to the laboratory via porters or air-tube systems. This approach is associated with delays such as; storing samples on ice to avoid increases in lactate due to *ex-vivo* anaerobic metabolism, transportation of samples to the laboratory and centrifugation before analysis leading to possible delays in the reporting of results to clinicians [6]. The central laboratory approach is also associated with prolonged vein-to-brain time -- the time it takes from blood draw to when the clinician becomes aware of the test results, resulting in a potential delay in clinical decision-making. As POC technology has advanced, near patient bench top blood gas analysers have been made available for lactate testing. However these devices are not portable and their availability is usually restricted to individual specialist units, e.g. Emergency Departments (ED) and Intensive Care Units (ICU). The advantage of blood gas analysers is that they have a more rapid turnaround time than central laboratory analysers [7]. However, sample turnaround time may be affected by delays in transportation to the ED or ICU, if the sample was drawn outside these major units.

Lactate measurement is one of the elements of the Sepsis Six and the Surviving Sepsis bundles [8]. The Survive Sepsis and Healthcare Improvement Scotland Sepsis campaigns have both recommended delivery of the Sepsis Six bundle within one hour of identification of patients with sepsis [9, 10]. Lactate measurement devices need to be placed at the point of need rather than in centrally-accessed locations to assist healthcare practitioners to achieve the one hour goal [7]. The potential benefits of POC handheld devices in the clinical setting are summarised in Table 1 [6, 8].

**Table 1:** Advantages of POC testing in clinical settings

Advantages of POC testing	
1) Simpler pre-analytical process	2) Requires small blood volume e.g. 95 μl
3) Allows bedside testing& portability	4) Provides rapid results
5) Accelerates clinical decision-making process	6) Allows healthcare practitioner to deliver patient-centred care
7) Decreases time to treatment	8) Potential to improve patients outcome

The aims of this study were: a) to compare lactate measurements of the i-STAT handheld device to near patient blood gas analysers situated in the ICU, and b) to determine the differential reporting times between the POC device and the near patient blood gas analysers in the ICU.

## Methods

### Instruments

The POC instruments compared in this study were: the i-STAT (Abbott Point Of Care, Princeton, USA) compared to the GEM Premier 4000 (Instrumentation Laboratory, UK) and the OMNI S (Roche Diagnostics, UK). Lactate assessment was performed by enzymatic reaction on all three POC analysers.

The i-STAT is a portable handheld device that measures blood gas levels at the bedside. It employs a single-use, disposable cartridge containing enzyme-coated biosensors. The CG4+ cartridge was used for lactate testing, according to the manufacturer’s instructions. The i-STAT requires 95 μL of blood to measure lactate, has a detection range from 0.30 to 20.00 mmol/L (according to the manufacturer) and determines lactate levels within 120 s. The i-STAT POC analysers are monitored automatically by internal quality control systems to monitor the integrity of the sample, sensors and fluidics with each use of cartridge.

The GEM Premier 4000 and OMNI S are compact, self-contained blood gas analysers that are located in the ICU and ED of our study sites. They both employ a multi-use cartridge containing enzyme-coated biosensors. The GEM premier 4000 and OMNI S have detection ranges of 0.10 to 20.00 mmol/L and 0.20 to 20.00 mmol/L lactate respectively. Our study sites blood gas analysers perform three types of automatic calibrations at different intervals. While those calibrations are running, testing cannot be performed leading to delays in analysing urgent blood samples. In addition, some blood gas analysers require a frequent replacement of the lactate electrode. The i-STAT employs a similar quality control system monitoring the quality of the cartridge, operators’ actions and the instrument performance.

### Study settings

This study was conducted at two large university affiliated district general suburban hospitals in Scotland (Royal Alexandra Hospital, Paisley and University Hospital Crosshouse). The Royal Alexandra Hospital [RAH] is a 650-bed hospital with a 7-bedded mixed medical and surgical closed Intensive Care Unit (ICU). The ICU admits approximately 350 adult patients a year (approx. 80 % of admissions require Level 3 critical care) [11]. At this hospital all lactate samples are measured using the blood gas analyser GEM Premier 4000.

University Hospital Crosshouse [UHC] is a 645-bed hospital with a 5-bedded mixed medical and surgical closed ICU. The ICU admits approximately 270 adult patients a year (approx. 70 % of admissions require Level 3 critical care) [11]. Within University Hospital Crosshouse, lactate samples are tested using the blood gas analyser OMNI S.

The acute care setting in the second stage of the study included both general medical and surgical wards as well as three 12-bedded high dependency units (HDU). The HDU within the RAH was an open mixed medical and surgical HDU admitting approximately 1400 patients per year. The two HDU’s within UHC were an open medical and an open surgical HDU admitting approximately 1100 and 700 patients accordingly [11].

### Study design

#### Patients

The study was approved by the ethical review board of the University of the West of Scotland. The need for patient consent was waived since the study was a service evaluation and all data were analysed anonymously. All of the patients were 16 years of age or older. Patients less than the age of 16 years of age were excluded from the study.

#### Methods

This study was conducted in two stages.

#### Method comparison stage

In the first stage, a method comparison for lactate was performed using 97 heparinised arterial blood samples from a total of 26 adult patients admitted to the ICUs of our study sites. The patients had a variety of different diagnoses requiring critical care. As this was a service evaluation, sampling and simultaneous measurement occurred at the discretion of the treating intensivist, who was not involved in the study. Samples were collected over a period of 2 weeks for each site. A single lot of i-STAT cartridges were used for each site. A single analysis was performed on the i-STAT and immediately thereafter (within median of 1–2 min for OMNI S and GEM 4000 respectively) analysed on the blood gas analyser of each hospital (GEM Premier 4000/ OMNI S). The analysers were run in accordance with their manufacturer’s recommendations. In this stage of the study, the same researcher, who was an appropriately trained laboratorian, performed all lactate testing at both sites.

#### Differential reporting times stage

In the second stage, a prospective observational study of samples of adult patients from different wards was conducted from January to July 2013. Advanced Nursing Practitioners (ANPs) were involved in this stage as they are the sepsis response team at the hospitals. The sepsis response team were triggered when a patient had an elevated early warning scoring system and clinical suspicion of infection. The ANPs at both sites were trained on the i-STAT before commencing the study. Lactate measurement is a clinical guideline at both hospitals for all patients with suspected infection. Therefore, there was no additional blood sample required for the purpose of this study. A heparinised venous sample was collected from patients suspected with infection. The sample was tested on the i-STAT at the patients’ bedside and then the standard procedure for lactate measurement was undertaken. The time impact of the i-STAT testing on the standard procedure for each hospital was taken into consideration during data analysis.

The time of blood draw, i-STAT testing start time, i-STAT results availability time, the starting and ending time of blood gas analyser testing and the time of blood gas analyser results availability at clinical area were recorded on data collection sheets. Then the data were entered into Microsoft Excel file (Microsoft Excel, 2010).

### Statistical analysis

Pearson correlation coefficient was used to determine the relationship between results from the POC handheld device and the blood gas analysers. The Bland-Altman method was used to determine the level of agreement between the analysers based on values of bias and limits of agreement [12].

The above tests provide the analytical difference between the analysers. However, to assess the clinical importance of the analytical difference between the analysers, patients were classified into three categories based on their lactate levels; low (<2.5 mmol/L), medium (2.5–3.99 mmol/L) and high (≥4 mmol/L). Lactate risk level classification was based on previous medical literature which linked lactate levels to patients’ outcome [13].

For the second stage of the study, differential reporting times were assessed using the Mann–Whitney test as the data were skewed. Accordingly, descriptive statistics were reported with quartiles for each site. All statistical and graphical analyses were performed using the GraphPad prism package (version 6.04, GraphPad Software Inc.). A prior power calculation was not conducted as this was a pilot study.

## Results

### Comparison of i-STAT against GEM premier 4000

**Fig. 1:**
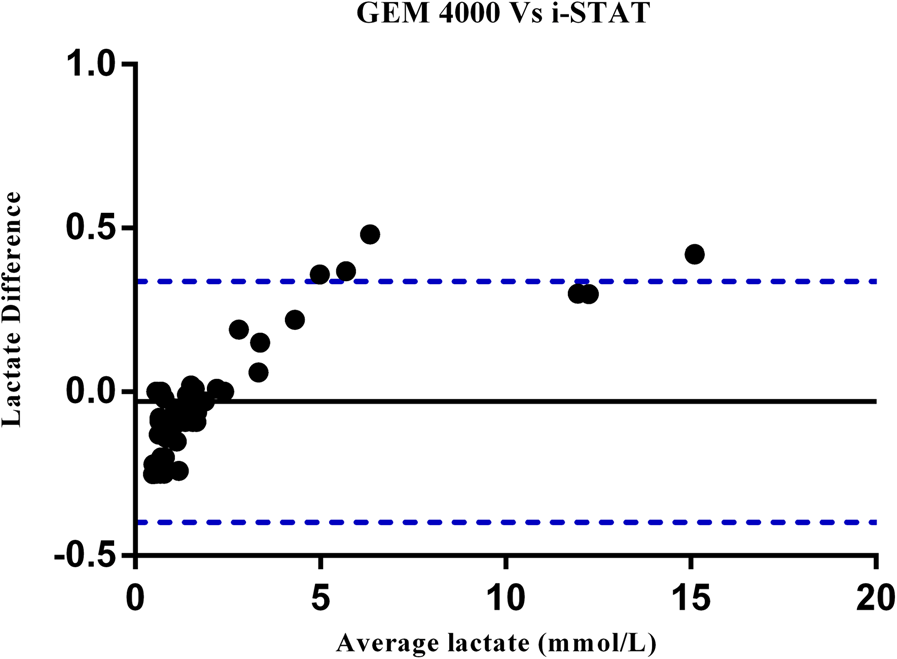
Bland-Altman plot of the difference between the lactate (mmol/L) measurements obtained by i-STAT and GEM 4000 analysers (calculated by i-STAT lactate measurements – GEM 4000 lactate measurements) versus the mean of the lactate measurements obtained by the two analysers. Solid line represents bias; dashed lines represent upper and lower 95 % limits of agreement

The Clinical Laboratory Standards Institute (CLSI) guidelines recommend a minimum of 40 observations in method comparison studies [14]. Therefore, 50 representative samples were analysed on the i-STAT and the GEM 4000 in parallel. Pearson correlation coefficient analysis showed a strong correlation between the i-STAT POC lactate and GEM 4000 analyser (*r* = 0.999). Bland-Altman statistics showed an average bias for the i-STAT of −0.03 lower than the GEM 4000 Premier with the 95 % limits of agreement ranging from −0.398 to 0.338 (Fig. 1).

Among the 50 samples (from 11 ICU patients) analysed on the GEM 4000 and the i-STAT; there were forty one samples classified as low, seven samples as medium and two samples as high lactate risk level categories. All samples (50) from the two analysers fell in the same lactate risk level categories (Table 2).

**Table 2:** Lactate risk category classification determined by the i-STAT and the blood gas analysers

Risk Category	i-STAT	GEM 4000	Risk Category	i-STAT	OMNI S
Low ≤2.5 mmol/L (*n* = 41)	41	41	Low ≤2.5 mmol/L (*n* = 45)	45	45
Intermediate 2.5–3.99 mmol/L (*n* = 7)	7	7	Intermediate 2.5–3.99 mmol/L (*n* = 2)	2	2
High ≥4 mmol/L (*n* = 2)	2	2	High ≥4 mmol/L (*n* = 0)	0	0

Legend: The above table shows that all lactate samples (50) analysed on the i-STAT and the GEM 4000 analysers fell in the same lactate risk level categories. Similarly, all lactate samples (47) analysed on the i-STAT and the OMNI S fell in the same lactate risk level categories

### Comparison of i-STAT against OMNI S

**Fig. 2:**
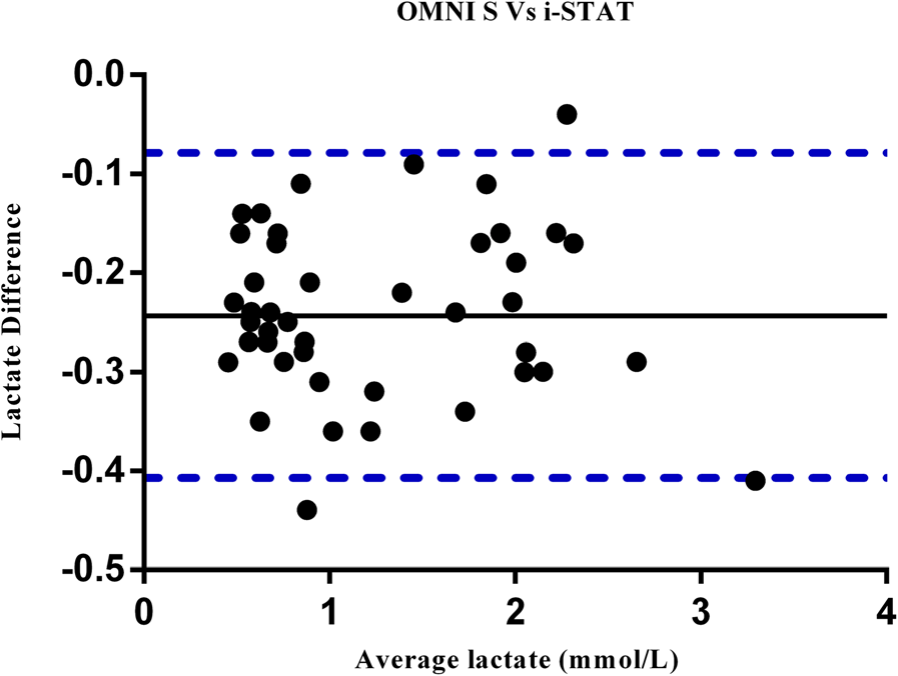
Bland -Altman plot of the differences between the lactate (mmol/L) measurements obtained by i-STAT and OMNI S analysers versus the mean of the lactate measurements obtained by the two analysers. Solid line represents bias; dashed lines represent upper and lower 95 % limits of agreement

Forty seven samples from 15 ICU patients were analysed on the i-STAT and OMNI S in parallel. Pearson correlation coefficient analysis showed a strong correlation between the i-STAT and OMNI S analyser (*r* = 0.993). Bland-Altman statistics showed an average bias for the i-STAT of - 0.24 lower than the OMNI S analyser with the 95 % limits of agreement ranging from - 0.407 to–0.079 (Fig. 2).

Among the 47 samples analysed on the OMNI S and the i-STAT; there were 45 samples classified as low, two samples as medium and zero samples as high lactate risk level categories. All samples (47) from the two analysers fell in the same lactate risk level categories (Table 2).

### Differential reporting times

A total of 136 lactate measurements were collected in this stage, but only 133 lactate results were available for statistical analysis (50 samples from 47 patients at the first hospital and 83 samples from 82 patients at the second hospital). Median time from blood draw to availability of the GEM 4000 blood gas analyser results at the clinical area was 10 min ([Q1–Q3 7.75–13]). At our second site, the median time from blood draw to availability of the OMNI S results at the clinical area was 11 min (Q1–Q3 8–22). Median time from blood draw to the i-STAT results was 5 min at both sites (Q1–Q3 5–7). The i-STAT was found to be significantly quicker at both sites (each *p-value* < 0.001). The Bland-Altman statistics in this stage showed an average bias for the i-STAT of −0.12 and −0.53 lower than the GEM 4000 and OMNI S respectively.

Interestingly, toward the end of the study, the nurses were informed that any lactate sample had to be sent to the laboratory for analysis due to a UK wide defect in the lactate module of the OMNI S. Consequently, the last eighteen of our study samples were sent to the central laboratory for analysis. This had led to an increase in the median time from blood draw to availability of the central laboratory results at the clinical area (median 133 min, Q1–Q 3 106–247). Those eighteen samples were also included in the final analysis of the above OMNI S data as this was a real life scenario which had an impact on lactate results availability.

## Discussion

Previous studies compared the i-STAT POC handheld device to central laboratory analysers with a strong correlation [13, 15]. However, our study is the first study to compare the i-STAT to near patient blood gas analysers for lactate. This study was conducted in two stages; method comparison and prospective observational. In the first stage of the study we compared the i-STAT POC handheld device to GEM 4000 and OMNI S near patient blood gas analysers located in the ICU of the study sites. Overall, our findings have shown that the i-STAT provides lactate results that are comparable with the hospitals’ blood gas analysers, as evidenced by the high Pearson correlation coefficient values and a small bias. The i-STAT lactate measurements were found to be lower than the GEM 4000 and OMNI S results. This is consistent with a false elevation of the lactate as the samples were collected without anti-glycolytic preservatives and tested on the blood gas analysers 1–2 min later after the i-STAT testing. The Bland-Altman bias was found to be higher in the second stage due to the prolonged time from blood draw to the analysis of the samples on the blood gas analysers (average of 7–8 min later of blood draw).

Our Bland-Altman figures have shown analytical variations in agreement across the range of the lactate measurements. However, to assess whether those differences are clinically acceptable we compared patients’ lactate risk level classification using the i-STAT and the comparative methods. We found the methods are generally comparable as lactate measurements from both analysers classified patients within the same lactate risk level. This shows that although there were small, clinically acceptable, analytical differences, this would not have affected clinical decisions.

In the second stage we determined the differential reporting times from blood draw to availability of lactate result at the clinical area. The findings from the second stage demonstrated that the i-STAT was significantly quicker than the near patient blood gas analysers in providing lactate results. Clinically, this can be translated into earlier identification of patients with high lactate levels and hence immediate clinical intervention. It also means that lactate testing can be repeated rapidly to determine lactate clearance and monitor patient response to resuscitation therapy.

While a time differential of 5–6 min for the lactate result may not initially appear to be clinically significant, a systems thinking approach would reveal that the analysis of the sample at the patient’s bedside allows for less opportunity for the sample to go missing as well as the fact that it provides the clinician with the time to care and to listen to their patient rather than take them away from the bedside to transport the specimen. Lactate measurement and result within an hour of recognition or presentation of sepsis is also a recognised international quality indicator for providing best care. Consequently, any marginal gain in providing a timely result has the potential to not only improve care and outcome but also avoid any financial disincentive for missing this target.

The sensor defect in the OMNI S blood gas analyser during the second stage reflects a real life situation within hospitals. This had a large impact on delaying lactate reporting times as the median time from blood draw to the lactate results increased to 133 min. Consequently, this may have had a negative impact on patients’ treatment and outcome.

Only a few studies have compared the differential reporting times of lactate POC to central laboratory analysers [16, 17]. Those studies were conducted in a different clinical setting and observed different time measurements to our study but their findings support the need for implementing POC testing in hospitals. Goyal et al. compared time from triage to POC lactate and time from triage to availability of the central laboratory results. The authors found that the use of POC lactate at triage decreased time to lactate result by a median of 151 min [16]. Similarly, Gaieski et al. compared differential reporting times between fingertip POC lactate and central laboratory lactate. The researchers found that the turnaround time of the fingertip POC lactate was shorter than the central laboratory’s turnaround time by a median of 65 min [17]. Those findings support our central laboratory time findings and show that central laboratory turnaround time may not be sufficient for early lactate measurement. The impact of using a handheld POC device on early initiation of sepsis treatment has not been widely researched. However, a recent study by Singer et al. reported that the use of POC testing in patients with sepsis reduced time to lactate results significantly (*p* < 0.001), time to intravenous (IV) fluids administration (*p* = 0.03) with a significant reduction in mortality (*p* = 0.02) and ICU admission (*p* = 0.02) [18]. Singer et al. later demonstrated that the use of POC testing in ED patients significantly reduced time to lactate results and other biochemical tests (*p* < 0.001). It also reduced time to completion of Computed tomography (CT) scanning in patients who received intravenous (IV) contrast (*p* = 0.04) and reduced ED length of stay in some patients [19]. Similarly, we believe that the use of lactate POC testing can assist healthcare practitioners to complete the Sepsis Six bundle within the target hour. Thus, critically ill patients are identified sooner and treated in a more timely manner.

The direct cost of POCT is found to be much higher than the costs of central laboratory testing. However, the use of portable POC handheld device has the potential to reduce length of hospital stay, accelerate patient flow in clinical settings and ultimately improve patient outcomes [20, 21]. In the context of sepsis, 100,000 patients, with an average cost of £20,000 per patient, are admitted to the UK hospitals annually due to sepsis [22]. The Parliamentary and Health Services reported recently that £4,000 can be saved per sepsis case if the basic principles of sepsis management are followed, namely the Sepsis Six [22]. Therefore, the cost of using POC testing in patients with sepsis could be outweighed by the long term impact on patients’ outcome. Evidence of cost-effectiveness analysis is required to explore the financial impact of handheld POC lactate testing in patients with sepsis.

Institutions that are unable to meet the recommended timeline for lactate measurement may benefit hugely from using POC handheld devices in their clinical settings. Pre-hospital or rural settings could also benefit from using lactate POC handheld devices to identify patients with increased lactate levels and increased predicted mortality. This would expedite the transfer of those patients to the hospital.

## Conclusion

In summary, we have shown that the i-STAT POC handheld device provides accurate, timely and efficient care thereby supporting the institute of medicine domains of quality healthcare. This was evidenced by our results that showed the i-STAT correlates strongly with the near patient blood gas analysers. We have also shown that the POC hand held device is significantly quicker than near patient blood gas analysers and laboratory analysers. We believe that the use of a POC handheld device facilitates immediate clinical decision making and initiation of therapies for sepsis patients such as administration of antibiotics, fluid resuscitation and escalation of care. Further studies on the impact of lactate POC testing on patients’ outcome are required.

## Key messages

- The i-STAT provides clinically acceptable lactate results
- POC handheld device provides lactate results significantly quicker than near patient blood gas analysers and laboratory analysers
- The use of a lactate POC handheld device has the potential to accelerate the decision-making process in patients with sepsis

## Abbreviations

POCT: Point of Care Testing
NHS: National Health Services
POC: Point Of Care
ED: Emergency Department
ICU: Intensive Care Unit
ANP: Advanced Nursing Practitioner
Q1: First quartile
Q3: Third quartile
CT: Computed Tomography
IV: Intravenous
