# Erratum to: The accuracy and timeliness of a Point Of Care lactate measurement in patients with Sepsis

**DOI:** 10.1186/s13049-016-0227-2

**Published:** 2016-03-31

**Authors:** Fatene Ismail, William G. Mackay, Andrew Kerry, Harry Staines, Kevin D. Rooney

**Affiliations:** Institute of Healthcare Policy and Practice, School of Health, Nursing and Midwifery, University of the West of Scotland, Paisley, UK; Institute of Healthcare Associated Infection, University of the West of Scotland and University Hospital Crosshouse, Kilmarnock, UK; Biochemistry Laboratory, Royal Alexandra Hospital, Paisley, UK; Sigma Statistical Services, Balmullo, UK; Intensive Care Unit, Royal Alexandra Hospital and Institute of Healthcare Policy and Practice, University of the West of Scotland, Paisley, UK

## Erratum

After publication of the original article [1], the authors noticed a mistake in Table 2. Patient numbers in lactate classification categories Low, Medium and High were incorrectly reported as forty one, seven and two respectively in the GEM 4000 column. The patient numbers that should have been reported in Table 2 are forty, three and seven. Table 2 appears in its correct form in this erratum.

**Table 2:** Lactate risk category classification determined by the i-STAT and the blood gas analysers

Risk category	i-STAT	GEM 4000	Risk category	i-STAT	OMNI S
Low <2.5 mmol/L (*n* = 40)	40	40	Low <2.5 mmol/L (*n* = 45)	45	45
Medium 2.5 – 3.99 mmol/L (*n* = 3)	3	3	Medium 2.5 – 3.99 mmol/L (*n* = 2)	2	2
High ≥4 mmol/L (*n* = 7)	7	7	High ≥4 mmol/L (*n* = 0)	0	0

The above table shows that all lactate samples (50) analysed on the i-STAT and the GEM 4000 analysers fell in the same lactate risk level categories

Similarly, all lactate samples (47) analysed on the i-STAT and the OMNI S fell in the same lactate risk level categories

Additionally, the incorrect patient numbers from Table 2 were reported in the ‘Results’ section. The ‘Comparison of i-STAT against GEM premier 4000’ sub-section should therefore have read as follows:

“Among the 50 samples (from 11 ICU patients) analysed on the GEM 4000 and the i-STAT; there were forty samples classified as low, three samples as medium and seven samples as high lactate risk level categories.”

This mistake does not alter the validity of the results and conclusions as all lactate samples analysed on the i-STAT and GEM 4000 analysers fell in the same lactate risk categories.
